# Evaluation of antithrombotic use and COVID-19 outcomes in a nationwide atrial fibrillation cohort

**DOI:** 10.1136/heartjnl-2021-320325

**Published:** 2022-03-10

**Authors:** Alex Handy, Amitava Banerjee, Angela M Wood, Caroline Dale, Cathie L M Sudlow, Christopher Tomlinson, Daniel Bean, Johan H Thygesen, Mehrdad A Mizani, Michail Katsoulis, Rohan Takhar, Sam Hollings, Spiros Denaxas, Venexia Walker, Richard Dobson, Reecha Sofat, the CVD-COVID-UK Consortium

**Affiliations:** 1 Institute of Health Informatics, University College London, London, UK; 2 University College London Hospitals National Health Service Trust, London, UK; 3 Barts Health National Health Service Trust, The Royal London Hospital, London, UK; 4 British Heart Foundation Cardiovascular Epidemiology Unit, Department of Public Health and Primary Care, University of Cambridge, Cambridge, UK; 5 British Heart Foundation Centre of Research Excellence, University of Cambridge, Cambridge, UK; 6 Health Data Research UK Cambridge, Wellcome Genome Campus and University of Cambridge, Cambridge, UK; 7 National Institute for Health Research Blood and Transplant Research Unit in Donor Health and Genomics, University of Cambridge, Cambridge, UK; 8 British Heart Foundation Data Science Centre, Health Data Research UK, London, UK; 9 Centre for Clinical Brain Sciences, University of Edinburgh, Edinburgh, UK; 10 Usher Institute, Edinburgh Medical School, University of Edinburgh, Edinburgh, UK; 11 UKRI Centre for Doctoral Training in AI-enabled Healthcare Systems, University College London, London, UK; 12 National Institute for Health Research University College London Hospitals Biomedical Research Centre, University College London, London, UK; 13 Institute of Psychiatry, Psychology and Neuroscience, King's College London, London, UK; 14 NIHR Maudsley Biomedical Research Centre, South London and Maudsley National Health Service Trust, London, UK; 15 Health Data Research UK London, University College London, London, UK; 16 MRC Unit for Lifelong Health and Ageing and Centre for Longitudinal Studies, University College London, London, UK; 17 National Health Service Digital, Leeds, UK; 18 British Heart Foundation Research Accelerator, University College London, London, UK; 19 Bristol Medical School: Population Health Sciences, University of Bristol, Bristol, UK; 20 Institute of Systems, Molecular and Integrative Biology, University of Liverpool, Liverpool, UK

**Keywords:** atrial fibrillation, COVID-19, epidemiology, electronic health records, drug monitoring

## Abstract

**Objective:**

To evaluate antithrombotic (AT) use in individuals with atrial fibrillation (AF) and at high risk of stroke (CHA_2_DS_2_-VASc score ≥2) and investigate whether pre-existing AT use may improve COVID-19 outcomes.

**Methods:**

Individuals with AF and CHA_2_DS_2_-VASc score ≥2 on 1 January 2020 were identified using electronic health records for 56 million people in England and were followed up until 1 May 2021. Factors associated with pre-existing AT use were analysed using logistic regression. Differences in COVID-19-related hospitalisation and death were analysed using logistic and Cox regression in individuals with pre-existing AT use versus no AT use, anticoagulants (AC) versus antiplatelets (AP), and direct oral anticoagulants (DOACs) versus warfarin.

**Results:**

From 972 971 individuals with AF (age 79 (±9.3), female 46.2%) and CHA_2_DS_2_-VASc score ≥2, 88.0% (n=856 336) had pre-existing AT use, 3.8% (n=37 418) had a COVID-19 hospitalisation and 2.2% (n=21 116) died, followed up to 1 May 2021. Factors associated with no AT use included comorbidities that may contraindicate AT use (liver disease and history of falls) and demographics (socioeconomic status and ethnicity). Pre-existing AT use was associated with lower odds of death (OR=0.92, 95% CI 0.87 to 0.96), but higher odds of hospitalisation (OR=1.20, 95% CI 1.15 to 1.26). AC versus AP was associated with lower odds of death (OR=0.93, 95% CI 0.87 to 0.98) and higher hospitalisation (OR=1.17, 95% CI 1.11 to 1.24). For DOACs versus warfarin, lower odds were observed for hospitalisation (OR=0.86, 95% CI 0.82 to 0.89) but not for death (OR=1.00, 95% CI 0.95 to 1.05).

**Conclusions:**

Pre-existing AT use may be associated with lower odds of COVID-19 death and, while not evidence of causality, provides further incentive to improve AT coverage for eligible individuals with AF.

## Introduction

Atrial fibrillation (AF) is a disturbance of heart rhythm affecting 37.5 million people globally^1^ and significantly increases the risk of stroke.^2^ Anticoagulants (AC), a subtype of antithrombotics (AT), reduce the risk of stroke^3^ and are recommended for individuals with AF and at high risk of stroke (CHA_2_DS_2_-VASc score ≥2, the National Institute for Health and Care Excellence (NICE) threshold).^4 5^ Despite improvements in AC uptake, previous evaluations suggest that up to one-third of individuals with AF and CHA_2_DS_2_-VASc score ≥2 in the UK may not be on AC,^6^ with around 15% on no type of AT.^6^ Hypotheses for this suboptimal medication centre around clinical overestimation of bleeding and fall risk in elderly patients,^6 7^ but the potential drivers of AT use remain underexplored at the population scale.

COVID-19 has presented another risk factor for individuals with AF, who are at increased risk of poor outcomes if they become infected.^8^ Observational evidence from Germany (n=6637) suggests that pre-existing AC use, but not antiplatelets (AP—another subtype of AT), may reduce mortality in individuals hospitalised with COVID-19.^9^ However, evidence is discordant, with a US study (n=3772) observing no difference in mortality in groups on AC or AP.^10^ In the UK, a larger study (n=70 464 of 372 746) explored AC and AC subtypes (warfarin vs direct oral anticoagulants (DOACs)) in individuals with AF and observed that AC was associated with lower COVID-19-specific mortality.^11^ This observational evidence is promising, but it does not compare all subtypes of AT and only covered the period up to 28 September 2020.

This study, therefore, set out to conduct the largest scale evaluation of AT use in individuals with AF to date in routinely updated, linked, population-scale electronic health record (EHR) data for 56 million people in England.^12^ Using this statistical power, this study investigated what factors are associated with pre-existing AT use and whether pre-existing AT use (across subtypes) is associated with COVID-19-related hospitalisation and death.

## Methods

### Study design and data sources

We conducted a cohort analysis using the newly established National Health Service (NHS) Digital Trusted Research Environment for England, which provides secure, remote access to linked, person-level EHR data for over 56 million people.^12^ Available data sources cover primary care, secondary care, pharmacy dispensing, death registrations and COVID-19 tests and vaccines. We used the General Practice Extraction Service Extract for Pandemic Planning and Research (GDPPR) for demographic and diagnostic data (eg, a diagnosis of AF) and the NHS Business Service Authority Dispensed Medicines (BSADM) for medication exposure data (eg, pre-existing AT use) as this is the most accurate available representation of the medication an individual takes. Hospital Episode Statistics (HES), COVID-19 Hospitalisations in England Surveillance System, Secondary Uses Service, and the Office for National Statistics (ONS) Civil Registration of Deaths were used for COVID-19 hospitalisation and death. Public Health England’s Second Generation Surveillance System was used to identify COVID-19 test results, and the COVID-19 vaccination events data set was used for COVID-19 vaccine status.

### Study populations

Individuals were included in the study if registered with a general practice (GP) in England (at least one record in the GDPPR data set with a valid person pseudo-identifier), ≥18 years old and alive on 1 January 2020, had available data on sex, ethnicity and GP location (based on the most recent, available data across primary care (GDPPR), secondary care (HES) and death registrations (ONS)), and had a diagnosis of AF (coded in GDPPR) with a CHA_2_DS_2_-VASc score ≥2 (calculated from the sum of components^13^ coded in GDPPR).

Individuals with contraindications to subtypes of AT (eg, DOACs in mitral stenosis, prosthetic mechanical valves, antiphospholipid antibody syndrome) were included as they are still eligible for other AT subtypes (eg, AP, warfarin).

To investigate exposure to pre-existing AT use on COVID-19-related hospitalisation and death, the inclusion criteria of a recorded COVID-19 event were applied. A COVID-19 event was defined as any positive test (PCR or lateral flow), a coded diagnosis in primary or secondary care, or a COVID-19 diagnosis on a death certificate (see Thygesen *et al* 14 and CALIBER^15^ for further details and phenotyping algorithms).

All phenotyping algorithms used are available on GitHub (https://github.com/BHFDSC/CCU020/tree/main/england/phenotypes) and online supplemental figure 1 provides a flow chart of individuals excluded at each stage.

10.1136/heartjnl-2021-320325.supp1 Supplementary data

### Study variables

#### Medication exposure

An individual was defined as taking a particular medication if they had one or more dispensed prescription (coded in the NHS BSADM) in the previous 6 months. We purposefully defined a liberal threshold to support evaluation of AT usage up to May 2021 that may have included unusual buying patterns (eg, bulk buying) caused by the pandemic.

Mutually exclusive medication categories were constructed for AC only, AP only, AP and AC, and no AT. Apixaban, rivaroxaban, dabigatran and edoxaban were collectively categorised as DOACs for comparison with warfarin. For analysis, three mutually exclusive medication categories were tested (any AT vs no AT, AC only vs AP only, DOACs vs warfarin).

### Outcomes

We defined two COVID-19 outcomes: COVID-19-related hospitalisation and COVID-19 death. COVID-19 hospitalisation included any hospital admission with a recorded COVID-19 diagnosis in any position (eg, not the primary diagnosis). COVID-19 death included individuals with a COVID-19 diagnosis on their death certificate in any position, a registered death within 28 days of their first recorded COVID-19 event or a discharge destination denoting death after a COVID-19 hospitalisation. Follow-up for COVID-19 outcomes ended on 1 May 2021, with the final follow-up date as either the date of the outcome of interest (eg, COVID-19 death) or the study end date (1 May 2021).

### Covariates

Covariates were preselected based on potential associations with pre-existing AT use^6^ or COVID-19 outcomes and included demographics (age, sex, ethnicity, geographical location, socioeconomic status, as measured by the Index of Multiple Deprivation decile), comorbidities that increase risk of stroke and bleeding (congestive heart failure, hypertension, stroke, vascular disease, diabetes, uncontrolled hypertension, renal disease, liver disease, prior major bleeding, hazardous alcohol use, history of fall, body mass index (BMI), smoking status) and other medications (antihypertensives, lipid-regulating drugs, proton pump inhibitors, non-steroidal anti-inflammatory drugs (NSAIDs), corticosteroids, other immunosuppressants and COVID-19 vaccination status, defined as at least one vaccine recorded in the COVID-19 vaccination events data set prior to the individual’s COVID-19 event).

The same covariates (excluding COVID-19 vaccination status) were used as independent variables to test associations with pre-existing AT use (for any AT vs no AT, AP only vs AC only, DOACs vs warfarin) and to calculate a propensity score for use as an additional covariate in the COVID-19 outcome analysis (as demonstrated in Elze *et al* 16).

### Statistical analysis

Descriptive statistics were used to summarise the study population characteristics and were stratified by medication category. Pairwise Pearson’s correlation coefficients were used to check for potential collinearities between covariates. Multivariable logistic regression was used to test associations with pre-existing AT use and calculate the propensity score.

Multivariable logistic regression and Cox regression were used to test differences between exposure groups (any AT vs no AT, AC only vs AP only, DOACs vs warfarin) for COVID-19-related hospitalisation and death. An additional post-hoc analysis compared dabigatran (a thrombin inhibitor) against factor Xa inhibitors (apixaban, edoxaban, rivaroxaban). Logistic and Cox regression methods were selected to evaluate potential differences between event-based (logistic regression) and time-to-event-based (Cox regression) analysis. All covariates including the propensity score were included in both methods (as demonstrated in Elze *et al* 16). For variables with incomplete data (BMI: 9.3% missing), individual values were imputed with the cohort mean.

Two sensitivity analyses were conducted. First, to evaluate the potential impact of different time periods, analysis was repeated for 1 January 2020–1 December 2020, prior to the introduction of vaccines and the 29 December 2020 cases peak of the second wave.^17^ Second, to validate the potential effect on COVID-19-specific outcomes, analysis was repeated with COVID-19 hospitalisation and death defined exclusively as the primary recorded diagnosis (coded first on hospital record or death certificate).

Primary results are reported from the multivariable logistic regression models covering the full time period (1 January 2020–1 May 2021), with the other analyses reviewed for concordance.

Data preparation was performed using Python V.3.7 and Spark SQL (V.2.4.5) on Databricks Runtime V.6.4 for Machine Learning, with analysis performed using R V.4.0.3. All codes for data preparation and analysis are available on GitHub (https://github.com/BHFDSC/CCU020/tree/main/england/code), with full results available at the following microsite: https://alexhandy1.shinyapps.io/at-evaluation-results/.

### Patient and public involvement

The UK National Institute for Health Research-British Heart Foundation (BHF) Cardiovascular Partnership lay panel comprising individuals affected by cardiovascular disease reviewed and approved this project.

## Results

### Evaluation of AT use

**Figure 1:**
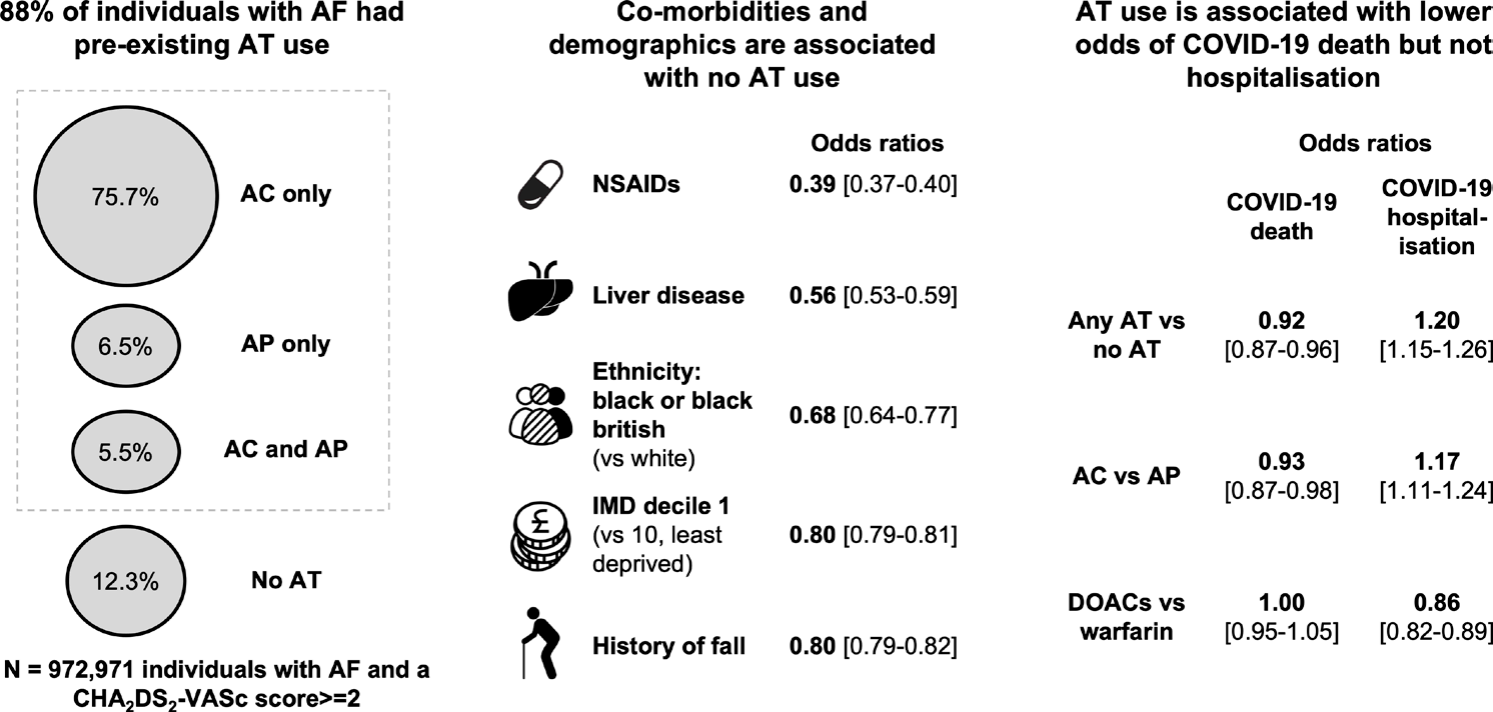
Visual overview of key study findings. AC, anticoagulants; AF, atrial fibrillation; AP, antiplatelets; AT, antithrombotics; DOACs, direct oral anticoagulants; IMD, Index of Multiple Deprivation; NSAIDs, non-steroidal anti-inflammatory drugs.

**Figure 2:**
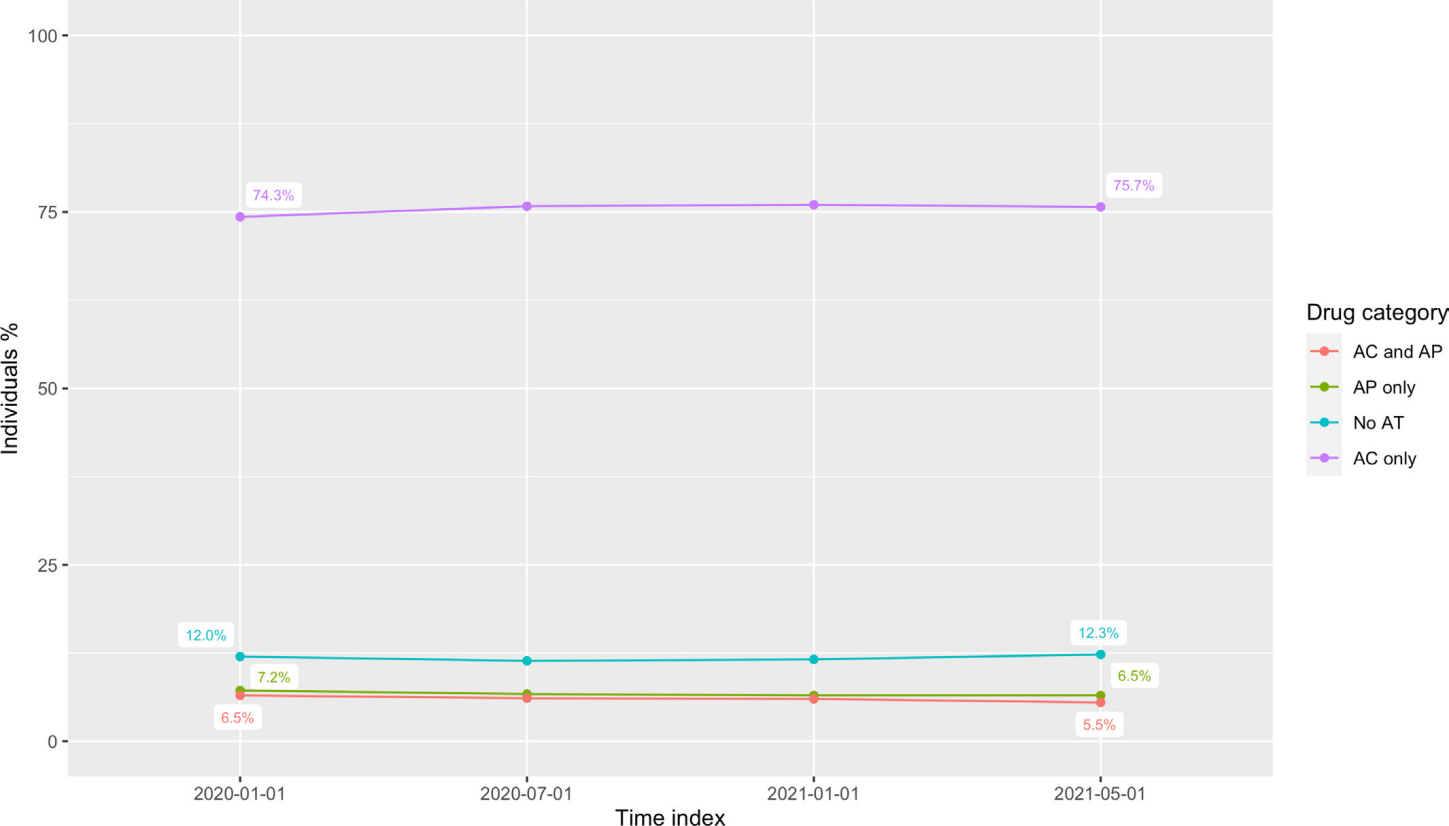
Individual antithrombotic prescriptions by drug category, January 2020–May 2021. AC, anticoagulants; AP, antiplatelets; AT, antithrombotics.

From a total of 55 903 113 individuals registered with a GP practice in England, 972 971 (1.7%) had a diagnosis of AF and a CHA_2_DS_2_-VASc score ≥2 on 1 January 2020 and 88.0% (n=856 336) had pre-existing AT use, with 74.3% (n=722 737) on AC only (see figure 1 for key study findings). The demographic and clinical characteristics of this cohort are summarised in tables 1–3. By May 2021, the proportion of individuals on any AT had fallen to 87.7%, but only AC had increased to 75.7% (see figure 2). For individuals on any AT, warfarin prescriptions fell from 24.8% in January 2020 to 17.1% in May 2021, while DOACs rose from 60.3% to 69.5% (see online supplemental figure 2).

**Table 1:** Study population demographic characteristics by antithrombotic medication category

	Total n (%)	Any AT n (%)	AC only n (%)	AP only n (%)	AC and AP n (%)	No AT n (%)
Individuals	972 971 (100)	856 336 (88)	722 737 (74.3)	70 498 (7.2)	63 101 (6.5)	116 635 (12)
Age, mean years (±SD)	79 (±9.3)	79 (±9)	79 (±8.9)	79 (±10)	78 (±8.9)	78 (±11)
65–74	229 464 (23.6)	198 956 (23.2)	166 943 (23.1)	16 018 (22.7)	15 995 (25.3)	30 508 (26.2)
≥75	686 578 (70.6)	610 497 (71.3)	518 205 (71.7)	49 702 (70.5)	42 590 (67.5)	76 081 (65.2)
Female	449 279 (46.2)	387 184 (45.2)	338 477 (46.8)	28 622 (40.6)	20 085 (31.8)	62 095 (53.2)
Ethnicity						
White	932 571 (95.8)	822 292 (96)	696 757 (96.4)	66 237 (94)	59 298 (94)	110 279 (94.6)
Asian or Asian British	20 557 (2.1)	17 699 (2.1)	12 797 (1.8)	2536 (3.6)	2366 (3.7)	2858 (2.5)
Black or black British	9418 (1)	7658 (0.9)	6200 (0.9)	862 (1.2)	596 (0.9)	1760 (1.5)
Mixed	3194 (0.3)	2636 (0.3)	2115 (0.3)	274 (0.4)	247 (0.4)	558 (0.5)
Other ethnic groups	7231 (0.7)	6051 (0.7)	4868 (0.7)	589 (0.8)	594 (0.9)	1180 (1)
Geographical locations						
South East	172 714 (17.8)	150 276 (17.5)	127 207 (17.6)	11 566 (16.4)	11 503 (18.2)	22 438 (19.2)
North West	143 391 (14.7)	127 860 (14.9)	106 990 (14.8)	10 705 (15.2)	10 165 (16.1)	15 531 (13.3)
East of England	104 591 (10.7)	92 676 (10.8)	78 194 (10.8)	7408 (10.5)	7074 (11.2)	11 915 (10.2)
South West	108 250 (11.1)	94 816 (11.1)	80 009 (11.1)	7863 (11.2)	6944 (11)	13 434 (11.5)
Yorkshire and the Humber	108 285 (11.1)	96 113 (11.2)	81 386 (11.3)	8405 (11.9)	6322 (10)	12 172 (10.4)
West Midlands	111 062 (11.4)	97 555 (11.4)	83 383 (11.5)	7836 (11.1)	6336 (10)	13 507 (11.6)
East Midlands	83 786 (8.6)	74 596 (8.7)	63 978 (8.9)	5773 (8.2)	4845 (7.7)	9190 (7.9)
London	95 746 (9.8)	81 824 (9.6)	66 815 (9.2)	7495 (10.6)	7514 (11.9)	13 922 (11.9)
North East	45 146 (4.6)	40 620 (4.7)	34 775 (4.8)	3447 (4.9)	2398 (3.8)	4526 (3.9)
IMD deciles						
1 (most deprived)	78 061 (8)	68 894 (8)	56 583 (7.8)	6490 (9.2)	5821 (9.2)	9167 (7.9)
10 (least deprived)	106 436 (10.9)	93 984 (11)	80 764 (11.2)	6795 (9.6)	6425 (10.2)	12 452 (10.7)

Percentages should be interpreted vertically for all variables, for example, proportion within category for variable, except for the first row showing percentage of individuals across AT medication categories.

AC, anticoagulants; AP, antiplatelets; AT, antithrombotics; IMD, Index of Multiple Deprivation.

**Table 2:** Study population comorbidities that increase the risk of stroke and bleeding by antithrombotic medication category

	Total n (%)	Any AT n (%)	AC only n (%)	AP only n (%)	AC and AP n (%)	No AT n (%)
CHA_2_DS_2_-VASc score components						
Vascular disease	169 797 (17.5)	159 892 (18.7)	103 946 (14.4)	23 815 (33.8)	32 131 (50.9)	9905 (8.5)
Stroke/TIA/thromboembolism	196 899 (20.2)	183 140 (21.4)	150 588 (20.8)	16 611 (23.6)	15 941 (25.3)	13 759 (11.8)
Congestive heart failure	247 562 (25.4)	228 877 (26.7)	192 023 (26.6)	15 038 (21.3)	21 816 (34.6)	18 685 (16)
Diabetes	268 437 (27.6)	242 060 (28.3)	197 216 (27.3)	21 602 (30.6)	23 242 (36.8)	26 377 (22.6)
Hypertension	675 676 (69.4)	600 623 (70.1)	505 514 (69.9)	49 678 (70.5)	45 431 (72)	75 053 (64.3)
CHA_2_DS_2_-VASc score, mean (±SD)	3.9 (±1.4)	4 (±1.4)	3.9 (±1.4)	4.1 (±1.5)	4.4 (±1.5)	3.4 (±1.3)
2	172 172 (17.7)	138 750 (16.2)	120 969 (16.7)	10 912 (15.5)	6869 (10.9)	33 422 (28.7)
3	245 979 (25.3)	213 057 (24.9)	184 241 (25.5)	16 289 (23.1)	12 527 (19.9)	32 922 (28.2)
4	252 047 (25.9)	224 255 (26.2)	190 707 (26.4)	17 875 (25.4)	15 673 (24.8)	27 792 (23.8)
5	162 318 (16.7)	149 105 (17.4)	122 356 (16.9)	12 995 (18.4)	13 754 (21.8)	13 213 (11.3)
≥6	140 455 (14.4)	131 169 (15.3)	104 464 (14.5)	12 427 (17.6)	14 278 (22.6)	9286 (8)
HAS-BLED score components						
Renal disease	315 940 (32.5)	284 379 (33.2)	237 965 (32.9)	24 423 (34.6)	21 991 (34.9)	31 561 (27.1)
Liver disease	8462 (0.9)	6707 (0.8)	5440 (0.8)	788 (1.1)	479 (0.8)	1755 (1.5)
Stroke	196 493 (20.2)	182 756 (21.3)	150 232 (20.8)	16 606 (23.6)	15 918 (25.2)	13 737 (11.8)
Major bleeding event	335 289 (34.5)	293 096 (34.2)	240 703 (33.3)	27 431 (38.9)	24 962 (39.6)	42 193 (36.2)
Harmful alcohol use	28 970 (3)	25 572 (3)	21 162 (2.9)	2274 (3.2)	2136 (3.4)	3398 (2.9)
Uncontrolled hypertension	66 576 (6.8)	58 873 (6.9)	48 444 (6.7)	5395 (7.7)	5034 (8)	7703 (6.6)
History of fall	119 738 (12.3)	103 615 (12.1)	85 718 (11.9)	10 717 (15.2)	7180 (11.4)	16 123 (13.8)
BMI, mean (±SD)	28.7 (±6)	28.8 (±6)	28.8 (±6.1)	28.1 (±5.6)	29 (±5.8)	27.9 (±5.9)
Smoking status (ever smoker)	638 775 (65.7)	566 860 (66.2)	472 208 (65.3)	48 567 (68.9)	46 085 (73)	71 915 (61.7)

Percentages should be interpreted vertically for all variables, for example, proportion within category for variable.

HAS-BLED score component bleeding medications excluded as it is measured within exposures and labile international normalized ratio excluded as it could not be accurately extracted from data sets.

AC, anticoagulants; AP, antiplatelets; AT, antithrombotics; BMI, body mass index; TIA, transient ischaemic attack.

**Table 3:** Study population characteristics of COVID-19 outcomes and other medications by antithrombotic medication category

	Total n (%)	Any AT n (%)	AC only n (%)	AP only n (%)	AC and AP n (%)	No AT n (%)
COVID-19 outcomes						
Any COVID-19 event	77 364 (8)	67 087 (7.8)	54 756 (7.6)	6743 (9.6)	5588 (8.9)	10 277 (8.8)
COVID-19 hospitalisation	37 418 (3.8)	33 150 (3.9)	26 887 (3.7)	3201 (4.5)	3062 (4.9)	4268 (3.7)
COVID-19 hospitalisation (primary diagnosis)	27 011 (2.8)	23 919 (2.8)	19 375 (2.7)	2319 (3.3)	2225 (3.5)	3092 (2.7)
COVID-19 death	21 116 (2.2)	18 173 (2.1)	14 553 (2)	2055 (2.9)	1565 (2.5)	2943 (2.5)
COVID-19 death (primary diagnosis)	15 297 (1.6)	13 158 (1.5)	10 522 (1.5)	1508 (2.1)	1128 (1.8)	2139 (1.8)
Other medications						
Antihypertensives	540 678 (55.6)	498 113 (58.2)	412 077 (57)	40 375 (57.3)	45 661 (72.4)	42 565 (36.5)
Lipid-regulating drugs	589 568 (60.6)	547 521 (63.9)	441 736 (61.1)	51 120 (72.5)	54 665 (86.6)	42 047 (36.1)
Proton pump inhibitors	409 429 (42.1)	369 461 (43.1)	286 984 (39.7)	39 180 (55.6)	43 297 (68.6)	39 968 (34.3)
NSAIDs	19 448 (2)	14 608 (1.7)	11 101 (1.5)	2317 (3.3)	1190 (1.9)	4840 (4.1)
Corticosteroids	80 347 (8.3)	71 706 (8.4)	59 511 (8.2)	5929 (8.4)	6266 (9.9)	8641 (7.4)
Other immunosuppressants	13 216 (1.4)	11 690 (1.4)	9498 (1.3)	1152 (1.6)	1040 (1.6)	1526 (1.3)
COVID-19 vaccine prior to COVID-19 event	9463 (1)	8248 (1)	6799 (0.9)	824 (1.2)	625 (1)	1215 (1)

Percentages should be interpreted vertically for all variables, for example, proportion within category for variable.

Pre-existing medication use was determined as ≥1 dispensed prescription in the 6 months prior to the cohort start date (1 January 2020).

AC, anticoagulants; AP, antiplatelets; AT, antithrombotics; NSAIDs, non-steroidal anti-inflammatory drugs.

**Figure 3:**
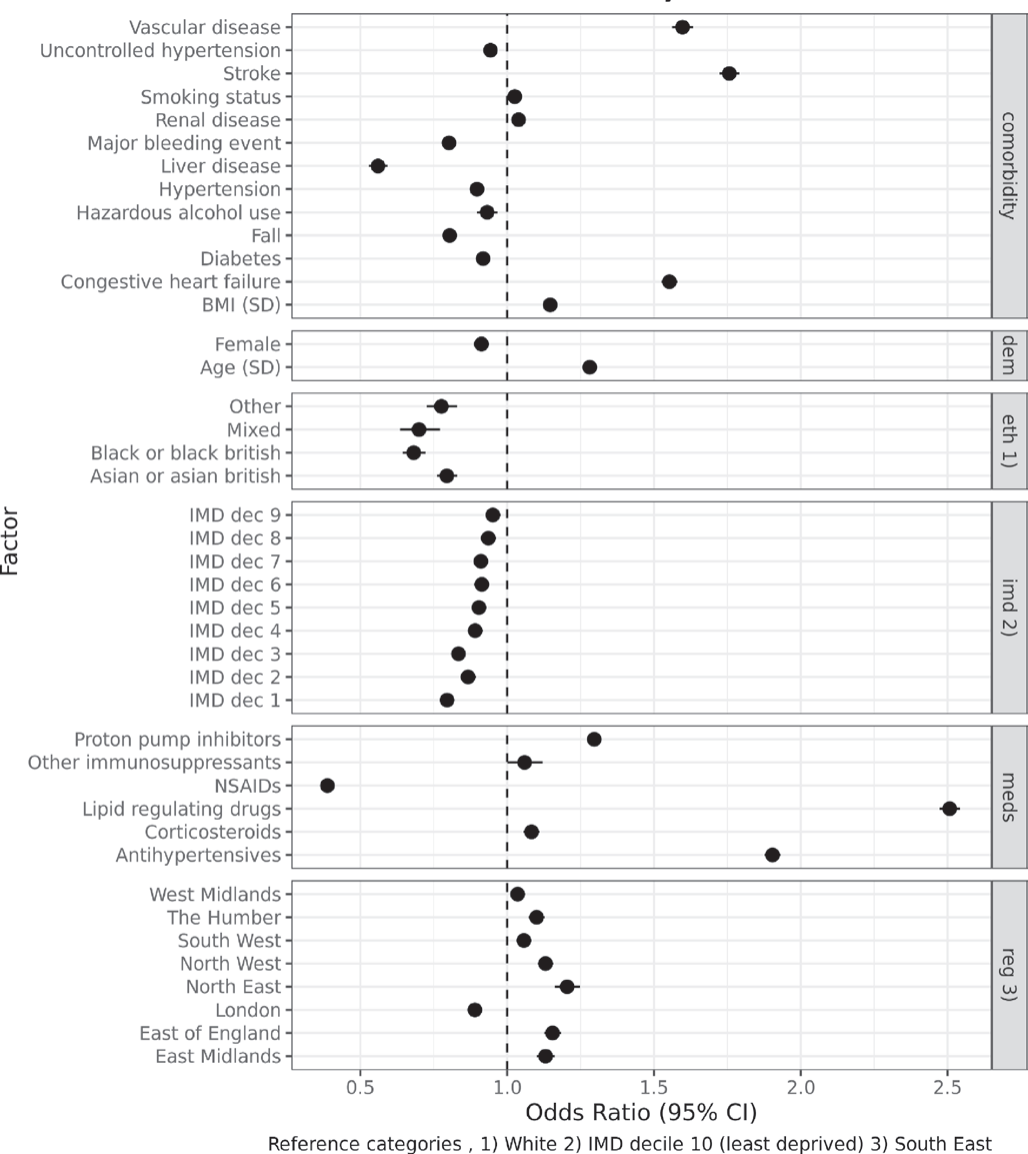
Factors associated with antithrombotics versus no antithrombotics (1 January 2020), using multivariable logistic regression. BMI, body mass index; IMD, Index of Multiple Deprivation; NSAIDs, non-steroidal anti-inflammatory drugs.

The factors associated with pre-existing AT use versus no AT are shown in figure 3. Lipid-regulating drugs (OR=2.50, 95% CI 2.47 to 2.54) and antihypertensives (OR=1.90, 95% CI 1.88 to 1.93) were associated with the highest odds of pre-existing AT use, followed by comorbidities in the CHA_2_DS_2_-VASc score (stroke: OR=1.76, 95% CI 1.72 to 1.79; vascular disease: OR=1.60, 95% CI 1.56 to 1.63). In contrast, NSAIDs (OR=0.39, 95% CI 0.37 to 0.40), liver disease (OR=0.56, 95% CI 0.53 to 0.59) and history of falls (OR=0.80, 95% CI 0.79 to 0.82) were associated with reduced odds.

Differences were also observed across demographics, ethnicity, socioeconomic status and geographical location, with women (OR=0.91, 95% CI 0.90 to 0.92) and individuals from ethnic minorities and lower socioeconomic positions associated with lower odds of AT use (eg, ethnicity of black or black British vs white; OR=0.68, 95% CI 0.64 to 0.72).

In other AT subtypes (AC vs AP and DOACs vs warfarin), the results were broadly consistent (see online supplemental figures 3 and 4), with the primary exception of vascular disease which was associated with reduced odds of AC versus AP (OR=0.37, 95% CI 0.36 to 0.38).

### AT use and COVID-19 outcomes

From 972 971 individuals who had a diagnosis of AF and a CHA_2_DS_2_-VASc score ≥2 on 1 January 2020, 8% (n=77 364) had a recorded COVID-19 event, 3.8% (n=37 418) had a COVID-19-related hospitalisation and 2.2% (n=21 116) died when followed up to 1 May 2021. The characteristics of individuals with a recorded COVID-19 event are summarised in online supplemental tables 1–3. Mean age (81) and comorbidities (mean CHA_2_DS_2_-VASc score 4.2) were both marginally higher compared with the full cohort. The proportion of individuals with pre-existing AT use was also marginally lower at 86.7%, but otherwise demographic and clinical characteristics were consistent.

**Figure 4:**
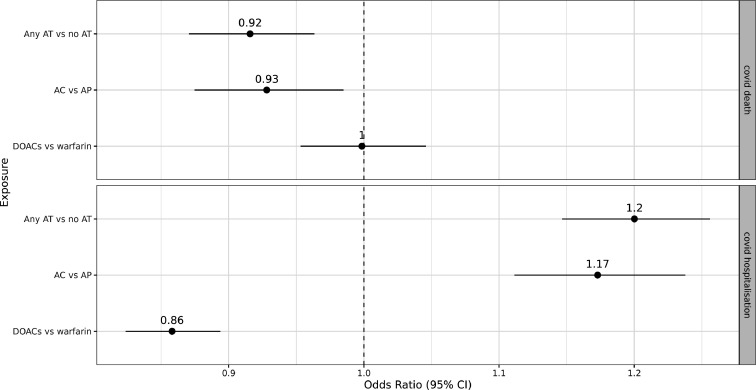
Comparison of AT medication exposures on COVID-19 outcomes (followed up to 1 May 2021) using propensity score adjusted multivariable logistic regression. AC, anticoagulants; AP, antiplatelets; AT, antithrombotics; DOACs, direct oral anticoagulants.

Pre-existing AT use was associated with lower odds of COVID-19 death (OR=0.92, 95% CI 0.87 to 0.96), but higher odds of COVID-19 hospitalisation (OR=1.20, 95% CI 1.15 to 1.26) (see figure 4). The same pattern was observed for AC versus AP (COVID-19 death: OR=0.93, 95% CI 0.87 to 0.98; COVID-19 hospitalisation: OR=1.17, 95% CI 1.11 to 1.24), but not for DOACs versus warfarin (COVID-19 death: OR=1.00, 95% CI 0.95 to 1.05; COVID-19 hospitalisation: OR=0.86, 95% CI 0.82 to 0.89). Dabigatran was associated with lower odds of COVID-19 death (OR=0.80, 95% CI 0.71 to 0.91) and hospitalisation (OR=0.88, 95% CI 0.79 to 0.98) compared with factor Xa inhibitors (see online supplemental figure 5).

These results were all directionally consistent across Cox regression analysis and the sensitivity analyses (see online supplemental figures 6–8).

Full results are available on the following microsite: https://alexhandy1.shinyapps.io/at-evaluation-results/.

## Discussion

### Main findings

In 972 971 individuals with AF and a CHA_2_DS_2_-VASc score ≥2, we observed 88.0% (n=856 336) with pre-existing AT use, which was associated with lower odds of COVID-19 death (OR=0.92, 95% CI 0.87 to 0.96). Although this association may not be causal, it provides further incentive to improve AT coverage for eligible individuals with AF.

Of the AF cohort analysed, 8% (n=77 364) had a recorded COVID-19 event, of which 3.8% (n=37 418) had a COVID-19-related hospitalisation and 2.2% (n=21 116) died. A marginally lower risk of COVID-19 death was observed for those with pre-existing AT use, which directionally aligns with the most comparable previous studies.^9 11^ AT use was, however, associated with higher odds of COVID-19 hospitalisation. This observation remained consistent when including only hospitalisations and deaths where COVID-19 was the first coded diagnosis. Higher observed risk of hospitalisation could reflect increased health-seeking behaviour (both patient-driven or by a clinician) of those with pre-existing AT use or may indicate that any risk reduction associated with AT use only materialises in the most serious cases. The same pattern was observed in AC versus AP and supports the findings of Fröhlich *et al* 9 that AC may be associated with lower risk of death than AP. For DOACs versus warfarin, no difference was observed between groups for COVID-19 death, but DOACs were associated with marginally reduced odds of COVID-19 hospitalisation. Our analysis did not directly investigate the previously reported observation that vitamin K depletion through warfarin is harmful,^18^ but more generally our findings suggest that it is unlikely that warfarin is associated with more severe COVID-19 outcomes compared with DOACs.^11^

Although these associations across AT subtypes do not prove causality, they provide further incentive to improve AT coverage for individuals with AF that are already at high risk of stroke. Previous evaluations in the UK have estimated that around 15% of these individuals do not take any AT and around 17% take AP only rather than the recommended AC.^3 6^ Our evaluation found around 12% on no AT and around 7% on AP only, which suggests national-level guidance^19^ and primary care incentives such as the Quality and Outcomes Framework^20^ continue to have a positive impact. Nonetheless, one in five individuals remain on a suboptimal medication regimen. Shifts from warfarin to DOACs observed in this study and others^21^ were recommended by COVID-19 guidance^22^ and demonstrate the potential impact of rapidly disseminated medications policy using population-scale EHR data.

Identifying which factors are associated with AT use is key to further lowering the proportion of individuals on suboptimal medication. NSAIDs displayed the strongest association with no AT use and likely reflects the association between NSAIDs and increased risk of major bleeding in individuals with AF.^23^ For comorbidities, liver disease had the strongest association with no AT use, which is also supported by clinical evidence.^24^ However, recent evidence suggests^25 26^ more personalised risk calculations for bleeding and stroke may enable more individuals with liver disease to benefit from AT. History of falls was the comorbidity with the second strongest association with no AT use, suggesting it remains a key factor in AT medicating decisions and may be overweighted as a proxy for bleeding risk.^7 27^ In the UK, NICE guidance was recently updated^4^ to explicitly address this issue and it will be important to track the impact of this in future evaluations. On demographics, lower odds of AT use were observed in women, but this is likely influenced by using NICE’s primary threshold for the CHA_2_DS_2_-VASc score of 2 for both sexes. The CHA_2_DS_2_-VASc score allocates 1 point to women and 0 for men, resulting in a larger proportion of comparatively healthy women (eg, 12% and 25% of women in the cohort have vascular disease and diabetes vs 21% and 33%, respectively, in men). However, demographic differences in AT use across ethnicity and socioeconomic status mirror systematic healthcare inequalities that have been reported previously.^28 29^ Targeted outreach to these groups will be key to improving AT use further.

### Strength and limitations

Routinely updated, linked, population-scale EHR data sets provide the statistical power to robustly analyse targeted subgroups and control for a wide range of potential confounders. The prevalence of individuals with AF and CHA_2_DS_2_-VASc score ≥2 in our cohort is similar to that observed in the Quality and Outcomes Framework,^20^ which provides an external validation for our data set. All code is open-source and an updated nationwide evaluation can be rapidly created for future time points.

The study does have limitations. First, the reported associations do not demonstrate causality and residual confounding is unlikely to have been fully eliminated. For example, in-hospital treatment regimens were not analysed so differences in COVID-19 outcomes due to additional targeted anticoagulation regimens^30^ or other medications cannot be accounted for in our analysis. While we attempted to mitigate confounding through careful cohort selection, covariates and propensity score adjustment, our study design does not control for all potential factors associated with the initiation of AT use which may influence COVID-19 outcomes. Second, our decision (supported by Elze *et al* 16) to include all covariates and the propensity score for the COVID-19 analysis could theoretically lead to overfitting; however, Elze *et al*’s^16^ own analysis demonstrates limited differences between methods. Lastly, exposure to AT medication was defined as one or more dispensed prescriptions (recorded in NHS BSADM) in the previous 6 months. Other studies have used different time periods and prescription frequency counts^9 11^ and adherence was not measured. We purposefully defined a liberal threshold to support evaluation of AT usage up to May 2021 that may have included unusual buying patterns (eg, bulk buying) caused by the pandemic. The trade-off is that for the COVID-19 outcome analyses it increases the probability of including a minority of ‘exposed’ individuals who had ceased regular, pre-existing AT medication.

## Conclusions

Pre-existing AT use may be associated with lower odds of COVID-19 death and, while not evidence of causality, provides further incentive to improve AT coverage for eligible individuals with AF.

**Key messages**

**What is already known on this subject?**

- Recent observational studies have shown that individuals routinely taking anticoagulants experienced less severe COVID-19 outcomes.
- These correlations are inconsistent across studies and have not compared all major subtypes of antithrombotics in one study.

**What might this study add?**

- Using routinely updated, linked electronic health record data for 56 million people in England, we were able to analyse antithrombotic use and their subtypes while controlling for a wide range of potential confounders.
- We identified 972 971 individuals with atrial fibrillation and a high risk of stroke (measured as CHA_2_DS_2_-VASc score ≥2) and observed 88.0% (n=856 336) with pre-existing antithrombotic use, which was associated with lower odds of COVID-19 death (OR=0.92, 95% CI 0.87 to 0.96).

**How might this impact on clinical practice?**

- These findings can help shape global antithrombotic medication policy and provide population-scale, observational analysis results alongside gold standard randomised control trials to help assess whether a potential beneficial effect of pre-existing antithrombotic use on COVID-19 death alters risk to benefit assessments in antithrombotic prescribing decisions.

## Data Availability

Data are available upon reasonable request. The de-identified data used in this study are available via the CVD-COVID-UK Consortium, coordinated by BHF Data Science Centre, for accredited researchers working on approved projects in NHS Digital’s TRE for England, but as restrictions apply they are not publicly available. The authors and colleagues across the CVD-COVID-UK Consortium have invested considerable time and energy in developing the data resource used here and are keen to ensure that it is used widely to maximise its value. For enquiries about data access, please see www.healthdatagateway.org/dataset/7e5f0247-f033-4f98-aed3-3d7422b9dc6d or email bhfdsc@hdruk.ac.uk.
