# Supplementary material for: Evaluation of antithrombotic use and COVID-19 outcomes in a nationwide atrial fibrillation cohort
Source: Heart. 2022 Mar 10;108(12):923–31. doi: 10.1136/heartjnl-2021-320325 (PMC8931797; doi:10.1136/heartjnl-2021-320325)
Supplement: Supplementary data [file heartjnl-2021-320325supp001.pdf]

Evaluation of antithrombotic use and COVID-19 outcomes in a nationwide atrial fibrillation cohort

SUPPLEMENTARY MATERIAL

SUPPLEMENTARY TABLES

Supplementary Table 1 - study population demographic characteristics for individuals with COVID-19 event by antithrombotic medication category
Percentages should be interpreted vertically for all variables e.g. proportion within category for variable, except for the first row showing percentage of individuals across AT medication categories.

	**Total**	**Any AT**	**AC only**	**AP only**	**AC and AP**	**No AT**
Individuals	77364 (100%)	67087 (86.7%)	54756 (70.8%)	6743 (8.7%)	5588 (7.2%)	10277 (13.3%)
Age (mean years, +/- sd)	81 (+/- 10.1)	81 (+/- 9.8)	81 (+/- 9.7)	82 (+/- 10.6)	79 (+/- 9.7)	81 (+/- 12)
65-74	12928 (16.7%)	11331 (16.9%)	9123 (16.7%)	1014 (15%)	1194 (21.4%)	1597 (15.5%)
>=75	59369 (76.7%)	51579 (76.9%)	42348 (77.3%)	5289 (78.4%)	3942 (70.5%)	7790 (75.8%)
Female	37227 (48.1%)	31498 (47%)	26569 (48.5%)	2992 (44.4%)	1937 (34.7%)	5729 (55.7%)
Ethnicity						
White	72745 (94%)	63132 (94.1%)	51899 (94.8%)	6162 (91.4%)	5071 (90.7%)	9613 (93.5%)
Asian or Asian British	2666 (3.4%)	2311 (3.4%)	1578 (2.9%)	389 (5.8%)	344 (6.2%)	355 (3.5%)
Black or Black British	1010 (1.3%)	844 (1.3%)	645 (1.2%)	118 (1.7%)	81 (1.4%)	166 (1.6%)
Mixed	281 (0.4%)	238 (0.4%)	187 (0.3%)	24 (0.4%)	27 (0.5%)	43 (0.4%)
Other Ethnic Groups	662 (0.9%)	562 (0.8%)	447 (0.8%)	50 (0.7%)	65 (1.2%)	100 (1%)
Geographical locations						

South East	11387 (14.7%)	9725 (14.5%)	8012 (14.6%)	913 (13.5%)	800 (14.3%)	1662 (16.2%)
North West	12691 (16.4%)	11153 (16.6%)	9024 (16.5%)	1113 (16.5%)	1016 (18.2%)	1538 (15%)
East of England	7095 (9.2%)	6163 (9.2%)	5047 (9.2%)	591 (8.8%)	525 (9.4%)	932 (9.1%)
South West	4185 (5.4%)	3577 (5.3%)	2911 (5.3%)	367 (5.4%)	299 (5.4%)	608 (5.9%)
Yorkshire and The Humber	8809 (11.4%)	7639 (11.4%)	6230 (11.4%)	878 (13%)	531 (9.5%)	1170 (11.4%)
West Midlands	13273 (17.2%)	11492 (17.1%)	9423 (17.2%)	1129 (16.7%)	940 (16.8%)	1781 (17.3%)
East Midlands	7279 (9.4%)	6439 (9.6%)	5376 (9.8%)	603 (8.9%)	460 (8.2%)	840 (8.2%)
London	8806 (11.4%)	7528 (11.2%)	5929 (10.8%)	802 (11.9%)	797 (14.3%)	1278 (12.4%)
North East	3839 (5%)	3371 (5%)	2804 (5.1%)	347 (5.1%)	220 (3.9%)	468 (4.6%)
IMD deciles						
1 (most deprived)	8396 (10.9%)	7331 (10.9%)	5843 (10.7%)	832 (12.3%)	656 (11.7%)	1065 (10.4%)
10 (least deprived)	6306 (8.2%)	5449 (8.1%)	4574 (8.4%)	480 (7.1%)	395 (7.1%)	857 (8.3%)

Supplementary Table 2 - study population comorbidities that increase stroke and bleeding risk for individuals with COVID-19 event by antithrombotic medication category

Percentages should be interpreted vertically for all variables e.g. proportion within category for variable

	**Total**	**Any AT**	**AC only**	**AP only**	**AC and AP**	**No AT**
CHA2DS2-VASc score components						
Vascular disease	169797 (17.5%)	159892 (18.7%)	103946 (14.4%)	23815 (33.8%)	32131 (50.9%)	9905 (8.5%)
Stroke / TIA / Thromboembolism	196899 (20.2%)	183140 (21.4%)	150588 (20.8%)	16611 (23.6%)	15941 (25.3%)	13759 (11.8%)
Congestive heart failure	247562 (25.4%)	228877 (26.7%)	192023 (26.6%)	15038 (21.3%)	21816 (34.6%)	18685 (16%)

Diabetes	268437 (27.6%)	242060 (28.3%)	197216 (27.3%)	21602 (30.6%)	23242 (36.8%)	26377 (22.6%)
Hypertension	675680 (69.4%)	600627 (70.1%)	505516 (69.9%)	49680 (70.5%)	45431 (72%)	75053 (64.3%)
CHA2DS2-VASc score (mean, +/- sd)	3.9 (+/- 1.4)	4 (+/- 1.4)	3.9 (+/- 1.4)	4.1 (+/- 1.5)	4.4 (+/- 1.5)	3.4 (+/- 1.3)
2	172174 (17.7%)	138751 (16.2%)	120968 (16.7%)	10914 (15.5%)	6869 (10.9%)	33423 (28.7%)
3	245977 (25.3%)	213057 (24.9%)	184242 (25.5%)	16290 (23.1%)	12525 (19.8%)	32920 (28.2%)
4	252051 (25.9%)	224259 (26.2%)	190710 (26.4%)	17874 (25.4%)	15675 (24.8%)	27792 (23.8%)
5	162315 (16.7%)	149102 (17.4%)	122353 (16.9%)	12996 (18.4%)	13753 (21.8%)	13213 (11.3%)
>=6	140457 (14.4%)	131171 (15.3%)	104466 (14.5%)	12426 (17.6%)	14279 (22.6%)	9286 (8%)
HAS-BLED score components						
Renal disease	315940 (32.5%)	284379 (33.2%)	237965 (32.9%)	24423 (34.6%)	21991 (34.9%)	31561 (27.1%)
Liver disease	8462 (0.9%)	6707 (0.8%)	5440 (0.8%)	788 (1.1%)	479 (0.8%)	1755 (1.5%)
Stroke	196493 (20.2%)	182756 (21.3%)	150232 (20.8%)	16606 (23.6%)	15918 (25.2%)	13737 (11.8%)
Major bleeding event	335289 (34.5%)	293096 (34.2%)	240703 (33.3%)	27431 (38.9%)	24962 (39.6%)	42193 (36.2%)
Harmful alcohol use	28969 (3%)	25571 (3%)	21161 (2.9%)	2274 (3.2%)	2136 (3.4%)	3398 (2.9%)
Uncontrolled hypertension	66576 (6.8%)	58873 (6.9%)	48444 (6.7%)	5395 (7.7%)	5034 (8%)	7703 (6.6%)
History of fall	119738 (12.3%)	103615 (12.1%)	85718 (11.9%)	10717 (15.2%)	7180 (11.4%)	16123 (13.8%)
BMI (mean, +/- sd)	28.7 (+/- 6)	28.8 (+/- 6)	28.8 (+/- 6.1)	28.1 (+/- 5.6)	29 (+/- 5.8)	27.9 (+/- 5.9)
Smoking status (ever smoker)	638774 (65.7%)	566861 (66.2%)	472208 (65.3%)	48568 (68.9%)	46085 (73%)	71913 (61.7%)

Supplementary Table 3 – study population characteristics for COVID-19 outcomes and other medications for individuals with COVID-19 event by antithrombotic medication category

Percentages should be interpreted vertically for all variables e.g. proportion within category for variable

	Total	Any AT	AC only	AP only	AC and AP	No AT
COVID-19 outcomes						
COVID-19 event	77364 (8%)	67087 (7.8%)	54756 (7.6%)	6743 (9.6%)	5588 (8.9%)	10277 (8.8%)
COVID-19 hospitalisation	37418 (3.8%)	33150 (3.9%)	26887 (3.7%)	3201 (4.5%)	3062 (4.9%)	4268 (3.7%)
COVID-19 hospitalisation (primary diagnosis)	27011 (2.8%)	23919 (2.8%)	19375 (2.7%)	2319 (3.3%)	2225 (3.5%)	3092 (2.7%)
COVID-19 death	21116 (2.2%)	18173 (2.1%)	14553 (2%)	2055 (2.9%)	1565 (2.5%)	2943 (2.5%)
COVID-19 death (primary diagnosis)	15297 (1.6%)	13158 (1.5%)	10522 (1.5%)	1508 (2.1%)	1128 (1.8%)	2139 (1.8%)
Other medications						
Antihypertensives	540681 (55.6%)	498116 (58.2%)	412078 (57%)	40377 (57.3%)	45661 (72.4%)	42565 (36.5%)
Lipid regulating drugs	589570 (60.6%)	547522 (63.9%)	441737 (61.1%)	51120 (72.5%)	54665 (86.6%)	42048 (36.1%)
Proton pump inhibitors	409430 (42.1%)	369462 (43.1%)	286984 (39.7%)	39181 (55.6%)	43297 (68.6%)	39968 (34.3%)
NSAIDS	19448 (2%)	14608 (1.7%)	11101 (1.5%)	2317 (3.3%)	1190 (1.9%)	4840 (4.1%)
Corticosteroids	80347 (8.3%)	71706 (8.4%)	59511 (8.2%)	5929 (8.4%)	6266 (9.9%)	8641 (7.4%)
Other immunosuppressants	13216 (1.4%)	11690 (1.4%)	9498 (1.3%)	1152 (1.6%)	1040 (1.6%)	1526 (1.3%)
COVID-19 vaccine prior to COVID-19 event	9463 (1%)	8248 (1%)	6799 (0.9%)	824 (1.2%)	625 (1%)	1215 (1%)

SUPPLEMENTARY FIGURES

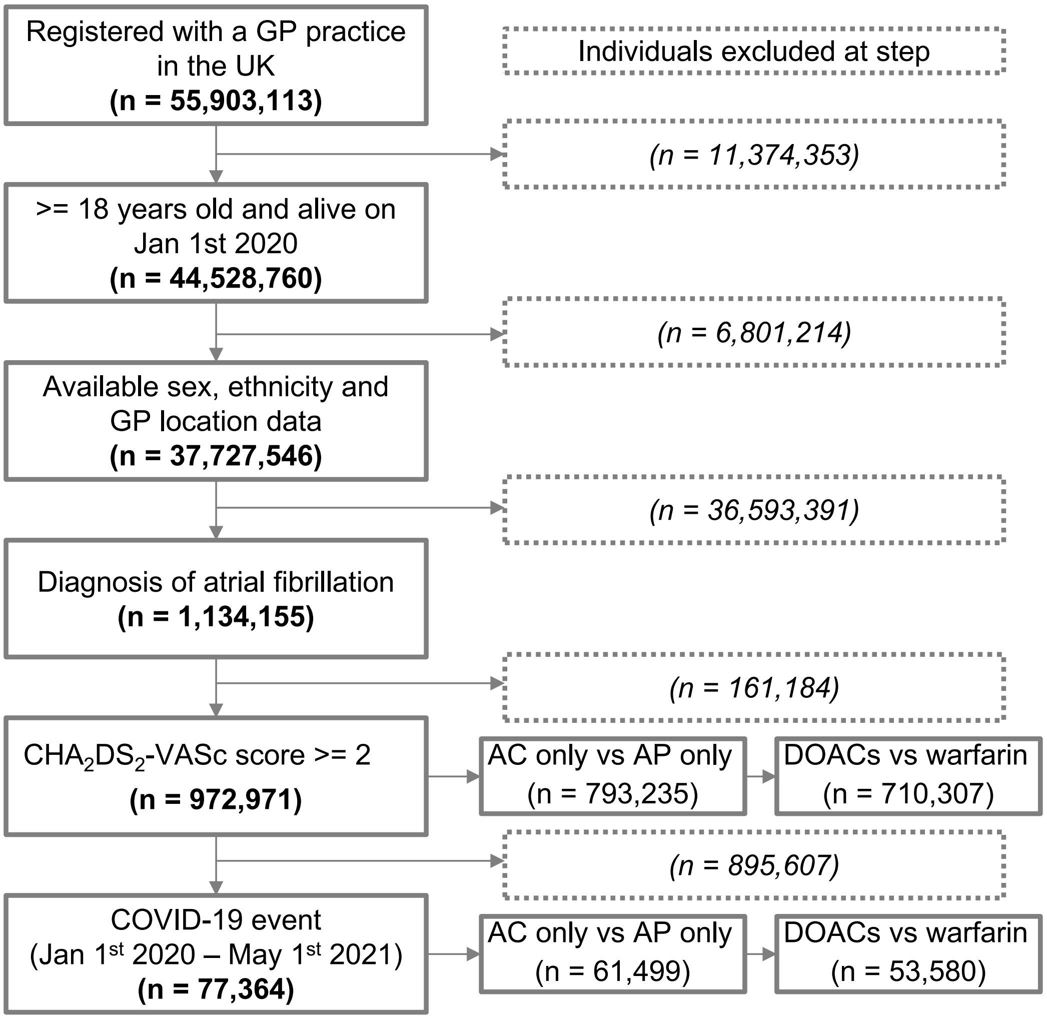

Supplementary Figure 1: Study flowchart showing the number of individuals excluded at each step and the study population sizes for each question

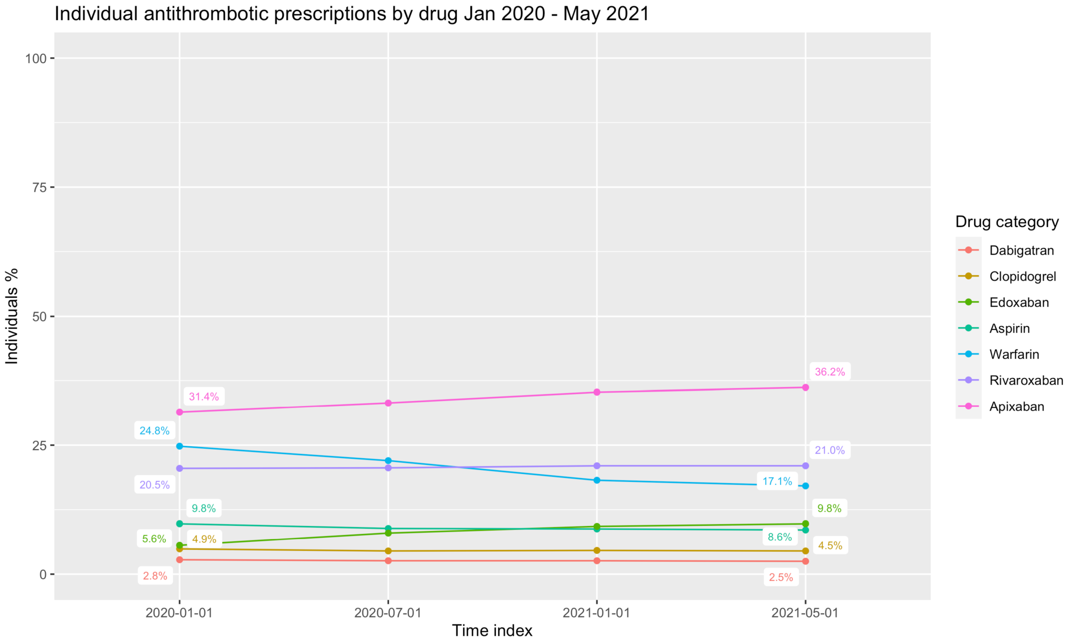

Supplementary Figure 2: Individual antithrombotic prescriptions by drug January 2020 – May 2021

Ordered by proportion of prescriptions with non-mutually exclusive categories e.g. an individual may have prescriptions for multiple drugs (warfarin and aspirin). Excludes drugs with <1% of prescriptions (ticagrelor, dipyridamole, prasugrel).

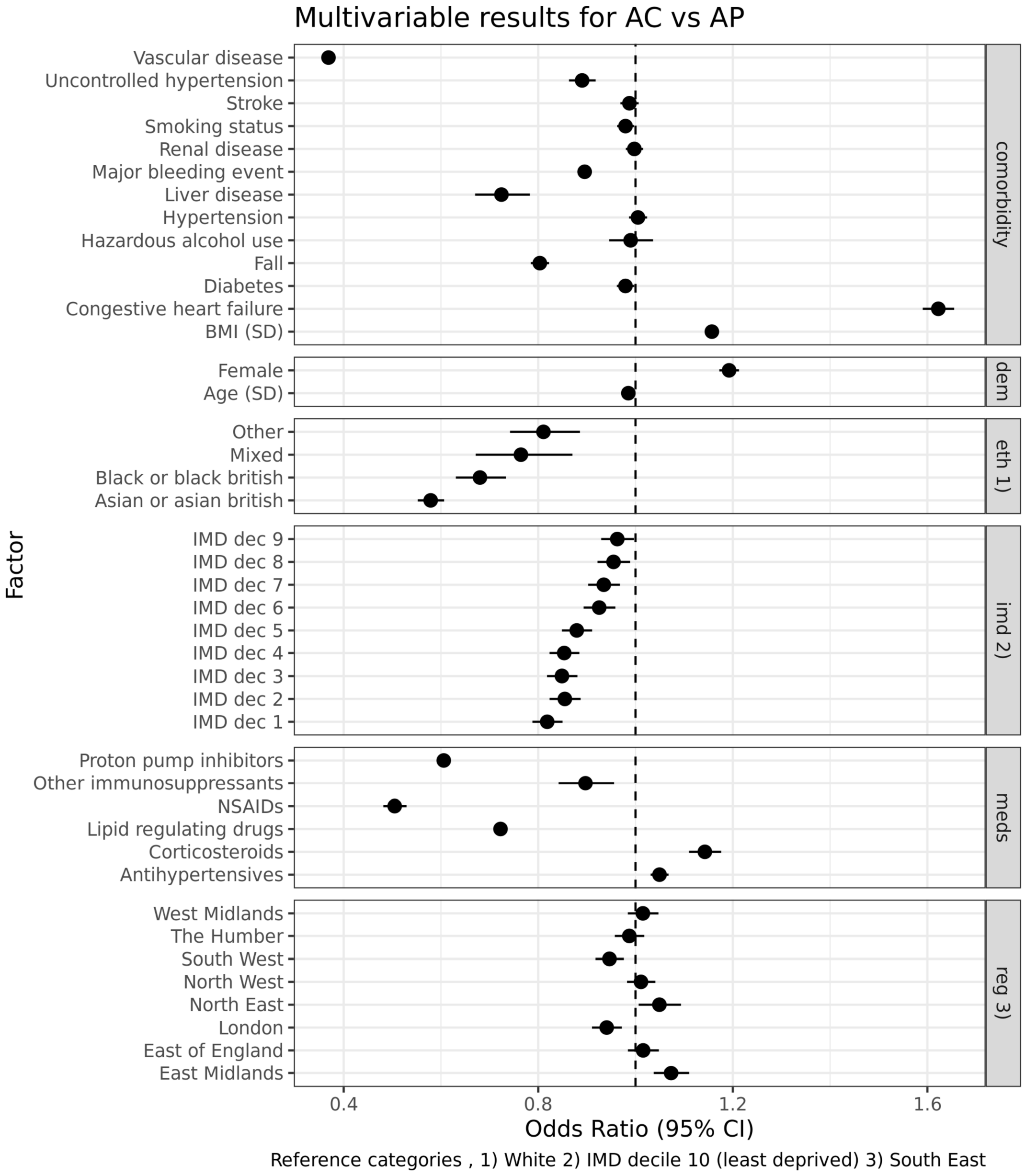

Supplementary Figure 3: Factors associated with AC vs AP (January 1st 2020) using multivariable logistic regression

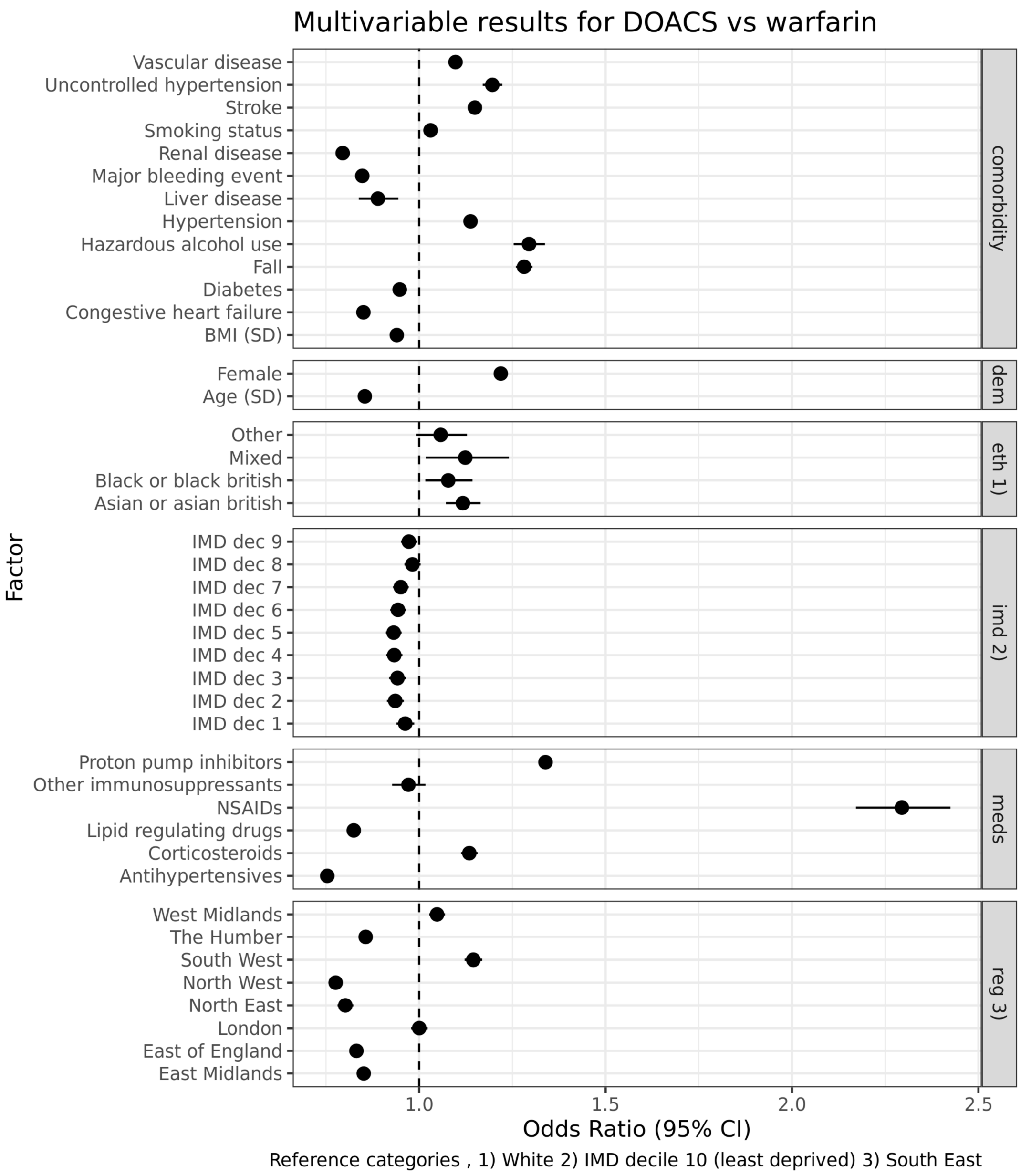

Supplementary Figure 4: Factors associated with DOACs vs warfarin (January 1st 2020) using multivariable logistic regression

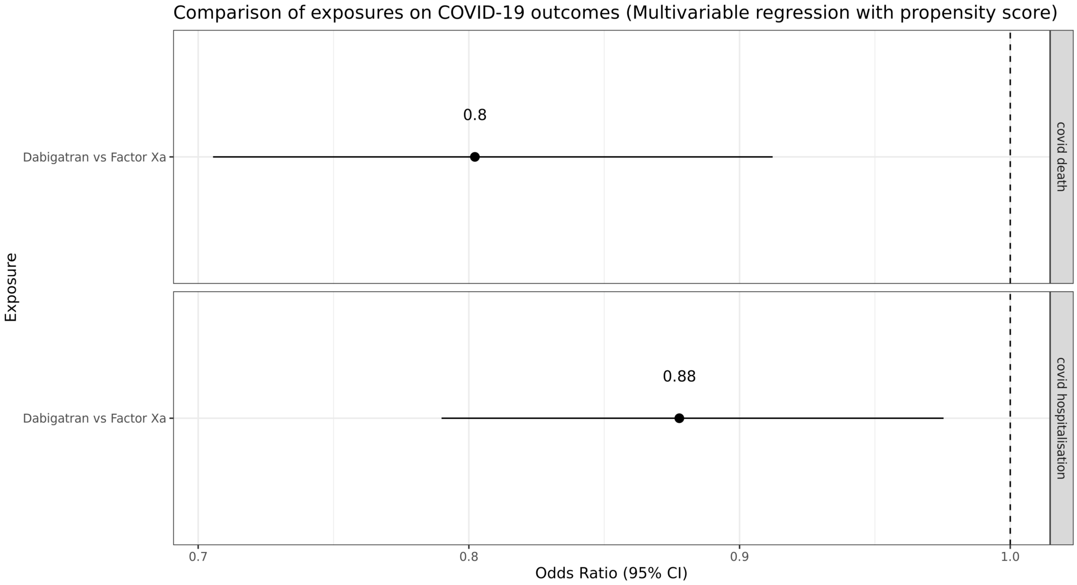

Supplementary Figure 5*: *Comparison of dabigatran versus factor Xa inhibitors on COVID-19 outcomes (follow up to May 1st 2021) using propensity score adjusted multivariable logistic regression

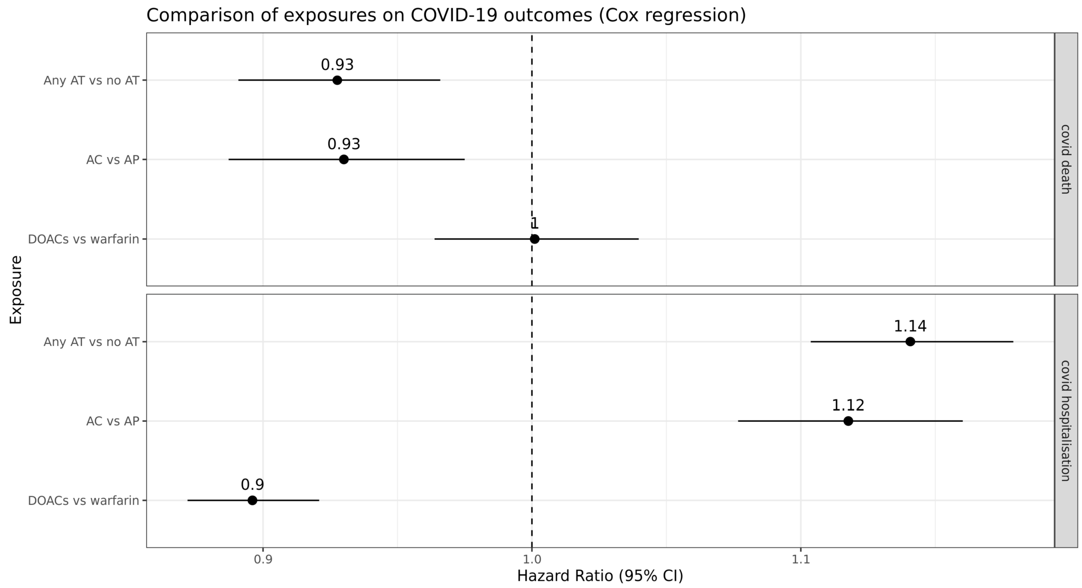

***Supplementary Figure 6*: Comparison of AT medication exposures on COVID-19 outcomes (follow up to May 1st 2021) using Cox regression**

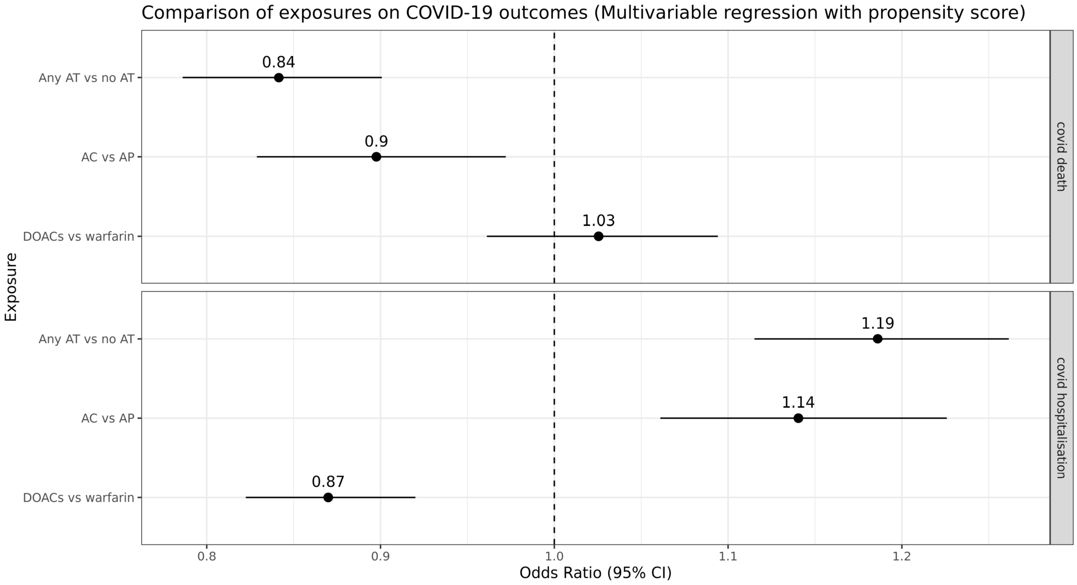

***Supplementary Figure 7*: Comparison of AT medication exposures on COVID-19 outcomes (follow up to December 1st 2020) using propensity score adjusted multivariable logistic regression**

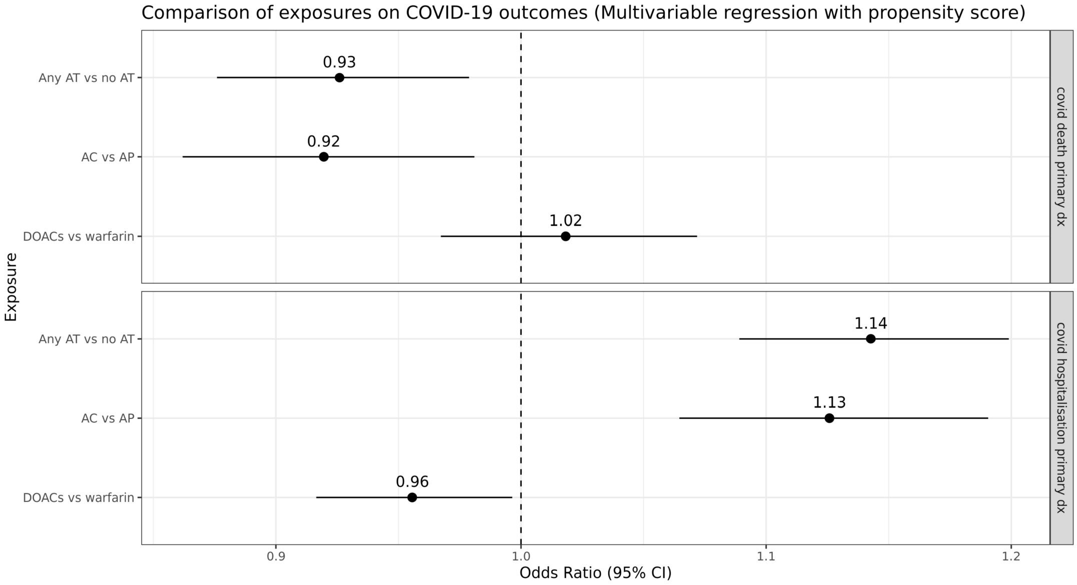

Supplementary Figure 8*: *Comparison of AT medication exposures on COVID-19 hospitalisation and death defined exclusively as the primary recorded diagnosis (follow up to May 1st 2021) using propensity score adjusted multivariable logistic regression
